# A New York Malpractice Case

**Published:** 1892-06

**Authors:** 


### A NEW YORK MALPRACTICE CASE.

A malpractice suit has just been tried against a physician of Fishkill Landing, New York, with the result of awarding a verdict of $2,500 damages against him. Some time since, a drayman fell from his cart and broke his arm. The bone protruded from the flesh, and the wound was filled with dirt. It was dressed and looked after by the physician, but the patient found it necessary to enter St. Luke's Hospital, New York, after about a month, at which time the arm was swollen to double its usual size. When he left the hospital, the arm was useless and will always remain so. Suit was therefore brought for malpractice, the claim being made that the wound was not properly cleansed. The damages were laid at $5,000 and a verdict given for $2,500.—*Buffalo Medical and Surgical Journal.*

